# Personalized Proteomics: The Future of Precision Medicine

**DOI:** 10.3390/proteomes4040029

**Published:** 2016-10-01

**Authors:** Trevor T. Duarte, Charles T. Spencer

**Affiliations:** Department of Biological Sciences, University of Texas at El Paso, El Paso, TX 79968, USA; ttduarte@utep.edu

**Keywords:** proteomics, personalized medicine, precision medicine, mass spectrometry, clinical proteomics, biomarker, diagnostic, pharmacokinetics, therapeutic monitoring

## Abstract

Medical diagnostics and treatment has advanced from a one size fits all science to treatment of the patient as a unique individual. Currently, this is limited solely to genetic analysis. However, epigenetic, transcriptional, proteomic, posttranslational modifications, metabolic, and environmental factors influence a patient’s response to disease and treatment. As more analytical and diagnostic techniques are incorporated into medical practice, the personalized medicine initiative transitions to precision medicine giving a holistic view of the patient’s condition. The high accuracy and sensitivity of mass spectrometric analysis of proteomes is well suited for the incorporation of proteomics into precision medicine. This review begins with an overview of the advance to precision medicine and the current state of the art in technology and instrumentation for mass spectrometry analysis. Thereafter, it focuses on the benefits and potential uses for personalized proteomic analysis in the diagnostic and treatment of individual patients. In conclusion, it calls for a synthesis between basic science and clinical researchers with practicing clinicians to design proteomic studies to generate meaningful and applicable translational medicine. As clinical proteomics is just beginning to come out of its infancy, this overview is provided for the new initiate.

## 1. The Age of Precision Medicine

Following the incorporation of the principles of Mendelian genetics into medicine, physicians interpreted non-infectious disease as the alteration in a single gene. Due to this alteration, a given disease could be simply treated in any presenting patient. Linkage of specific diseases to their genetic alteration became routine, e.g., mutation of Cystic Fibrosis Transmembrane Conductance Regulator *(cftr)* leading to cystic fibrosis, hemoglobin S causing sickle cell disease, BRCA mutations causing breast cancer, etc [1]. Despite the recognition of inherent uniqueness amongst the human population, this one disease—one treatment paradigm persisted with no better option.

As treatments continued, observations of the variable response to medications emerged ranging from full effective treatment, to little or no benefit, to severe adverse events. In addition, the influence of epigenetic and environmental factors was recognized to cause diverse presentation of a single disease. These diseases were labeled as multifactorial diseases to distinguish them from the simple conditions attributable to single genetic deficiencies. The management of these multifactorial diseases highlighted the need for quantitation of the influence of genetics, physiology, epigenetics, and environment on disease progression and treatment options for individual patients.

The first step toward this level of specificity was the completion of the Human Genome Project in 2003. This allowed for two key discoveries that have led to the age of genomic medicine; single nucleotide polymorphism (SNP) and the microarray analysis used to detect them [2,3]. SNPs account for about 90% of known genetic polymorphisms in the 0.9% of our genome that makes each individual unique [2]. Characterization of SNPs in variegated pathologies and treatment success have associated different molecular signatures with the diagnosis, prognosis, and therapy given to individual patients. This led to an abundance of genetic variation profiling in disease susceptibility and response to treatment centralized with the International HapMap Project [4]. Subsequent technological advances have plummeted the cost of sequencing the human genome from the $3 billion required by the Human Genome Project to just $1500 [5]. This has allowed for the complete sequencing of thousands of human genomes by the 1000 Genomes Project [6,7,8,9], which at last count contained over 2500 individuals [10]. A patient’s entire genetic profile can now readily be sequenced and risk factors for disease susceptibility, treatment efficacy, and adverse events identified allowing a physician to treat patients based upon their individual genetic makeup. Since one’s genome is relatively immutable, once a patient’s genome is sequenced the predisposition for any and all associated diseases could be determined. To protect against discrimination based upon use of genetic information, the Genetic Information Nondiscrimination Act (GINA) [11] was passed in 2008, paving the way for routine sequencing of patient genomes and genomic medicine.

Despite the level of detail provided by a genome sequence, this only illuminates one component contributing to multifactorial diseases. Indeed, the Human Genome Project revealed about ~21,000 protein coding genes (~3% of the genome) leaving 97% of the genome innocuous. However, further mechanistic studies into this junk DNA uncovered a plethora of regulation through interactions with both protein and RNA indexed by the Encyclopedia of DNA Elements (ENCODE) project [12,13]. These studies revealed 4 million locations within our genome that serve as switches to control the transcriptional activity of the ~21,000 genes. While much has been learnt and many lives improved thanks to genomic medicine, genetics cannot predict the diversity of protein expression patterns, posttranslational modifications (PTMs), or protein-protein interactions that control an individual’s response to disease or treatment.

While, precision medicine has the same roots as genomic medicine, it goes far beyond genetics taking into account the full complexity of cellular physiology [14]. Due to the dynamic nature of the proteome, PTMs and the interactome, personalized proteomics is fluid, adapting to individuals and individual situations, e.g., the proteins expressed by tissues during infection are not the same as those expressed prior to infection, after infection, or in uninfected persons [15,16,17,18]. Therein, Precision Medicine seeks to incorporate an individual’s cellular physiology, environment and medical history to create a custom treatment plan unique to each individual for each condition they experience.

In order to generate this holistic view, analytical technology evolved from analysis of a single biomolecule to a diverse collection of analytes that together can interpret a patient’s physiological state. The development and use of RNA microarrays allowed not only for determination of gene expression but also associations between the expressions of numerous proteins with disease states. The subsequent evolution of protein, peptide, and small molecule-based arrays increased the diversity of biomolecules and functions analyzable by microarray, e.g., expression, kinome and interactome profiling [19]. Yet microarrays are still limited by the array printed on the chip, an array that must be predetermined prior to production.

At the cellular level, flow cytometric analysis has become virtually ubiquitous *in clinica* particularly for determining the state of the immune response against infectious diseases and in cancer diagnosis. The limitations of spectral separation were partially overcome by combining a flow analyzer with a mass spectrometer to yield the mass cytometer (CyTOF) [20]. Despite the subsequent rise in the number of markers analyzed per cell, it is yet still limited at ~40 for top-of-the-line instruments. Together, mass spectrometry offers the most holistic, integrated system for clinical analysis of patient samples, seeking both known and unknown biomolecules (Table 1). Mass spectrometers can analyze proteins, peptide fragments, small molecules, antibodies, metabolites, and lipids. This collection, obtainable from a single platform, can generate the sum total of a patient’s physiological state needed for quick and proper diagnosis, exact treatment selection, and therapeutic monitoring making proteomics the future of precision medicine.

## 2. Clinical Proteomic Technology

Although the field of proteomics offers a great deal of promise in these areas, few assays or valid markers have made their way into the clinical laboratory [21,22,23,24,25]. The reasons for discrepancy between basic and clinical research are in part due to the complexity of the analyses and the variety of means to accomplish it. The new field of clinical mass spectrometry proteomics (cMSP) [26] seeks to unify disparate basic science approaches and validate them for clinical proteomics [21].

**Scheme 1:**
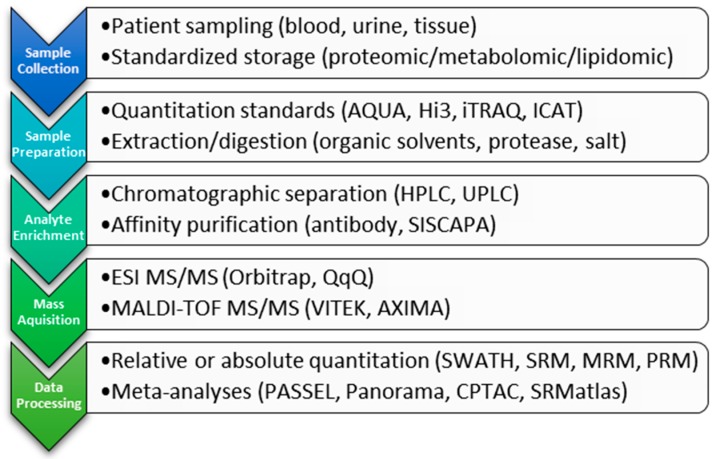
Workflow of clinical proteomic steps highlighting and placing in context examples from the text.

Proteomic studies involve a combination of target enrichment, ionization, detection, and quantitation. Samples derived from tissue, plasma, serum or urine are complex and currently are depleted of complexity or enriched for a specific analyte of interest prior to analysis (Scheme 1). Methods to achieve this separation include the use of antibody purification [27,28] or chromatographic separation (LC, UPLC) either in normal or reverse phase [29]. In the clinical laboratory, the method(s) used will depend upon the goal of the analysis, taking into consideration the costs and benefits associated with each methodology, reviewed in [21,24,25]. As technology advances, this pre-processing may become obsolete, reducing the time to analysis without compromising sensitivity and accuracy.

Throughout the years, various mass spectrometric methods have been developed for clinical proteomics [25]. That being said, two mass spectrometry methods have stood the test of time: Matrix Assisted Laser Desorption Ionization-Time of Flight mass spectrometry (MALDI-TOF MS) and liquid chromatography electrospray ionization tandem mass spectrometry (LC-ESI-MS/MS). Mass spectrometers are distinguished by ionization technique employed and the mass detector used to report the data.

### 2.1. Ionization

Although a number of ionization methods have been developed to analyze clinically relevant biomolecules, two are commonly regarded as most appropriate for the detection of large molecules: electrospray ionization (ESI) [30] and Matrix Assisted Laser Desorption Ionization (MALDI) [31]. These soft ionization techniques leave the molecules intact following ionization.

MALDI employs a low energy laser to ionize macromolecules which have been co-crystallized within a matrix usually composed of organic acid. The laser is absorbed by the matrix, causing a rapid gas phase expansion. The co-crystallized macromolecule is expelled as an analyte. The ionized analyte is then passed to the detector via manipulation through a magnetic field.

In ESI, analytes in aqueous phase are subjected to a high difference of potential between the emitter tip and the mass spectrometer instrument. The ions now in gas phase enter the instrument and are directed to the detector. ESI detectors range from low resolution triple quadrupole instruments to high resolution Orbitrap mass spectrometers.

### 2.2. Detection

#### 2.2.1. Triple Quadrupole

Quadrupole instruments are composed of four poles that are subjected to an alternating radio frequency (RF) field. This RF field focuses ions as they travel to the detector and can act as either a filter to select for a specific mass/charge (*m*/*z*) range or as a collision cell for fragmentation of a specific parent *m*/*z* ion. In the triple quadrupole (QqQ) instrument Q1 and Q3 are used as filters and Q2 is used as a collision cell, where an electric potential is applied to fragment the analyte of interest. This ability of the triple quad to filter a specific parent ion, fragment it and then filter for a specific product ion is the basis for the most widely used quantitative methods for targeted proteomics, select reaction monitoring (SRM) and multiple reaction monitoring (MRM). Given the complexity of biological samples, chromatographic separation can still lead to elution of *m*/*z* species of vastly different abundance. This overlap results in the suppression of some ions and a reduction in sensitivity for the mass spectrometric detection. To compensate for this, specific precursor ion fragments and their products after fragmentation are identified and targeted for analyses [32,33]. SRM is ideal for quantitation of peptides when used in combination with stable isotope labeled tryptic digests [34] or for top-down analyses when small proteins with known mass fragmentation are being investigated. This method is subject to reduced specificity when very complex samples are analyzed; therefore, extensive fractionation techniques are employed which may not be suitable for high-throughput requirements of clinical laboratories. Improvements upon this have been achieved through increased separation by ultra-high pressure LC, the use of high resolution mass spectrometers (see below), and the utilization of a hybrid ion trap/triple quadrupole mass spectrometer. In this method (MRM^3^), quantitation is based upon the production of a second generation multistage mass spectrometric (ms^3^) fragment and has been used to quantitate the inflammatory markers C-reactive protein and metalloproteinase inhibitor-1 from plasma samples [35].

#### 2.2.2. High-Resolution, Mass-Accurate Spectrometers

Overcoming sample complexity can also be achieved through the use of high mass accurate instruments. These state-of-the-art detectors have high resolutions over a broad mass range and mass accuracies below 5 parts per million. This translates to mass values accurate to the fourth decimal place. Two categories of high resolution mass spectrometers are well suited to clinical laboratories, the time of flight (TOF) MS coupled to MALDI ionization and the Orbitrap mass analyzer (OT ESI-MS/MS) either as stand-alone [36] or in combination with a triple quadrupole instrument q-OT ESI MS/MS [37].

TOF instruments pass ions through a field free tube to a detector and assign *m*/*z* values according to the time it takes for an ion to arrive at the detector. The addition of a quadrupole on the front end allows for higher resolution and when operated with small extraction windows qTOF MS is routinely used for data independent mass analyses. These powerful instruments provide higher selectivity and are only slightly less sensitive than triple quadrupole instruments operating in SRM without the required cumbersome optimization [38]. The versatility of the MALDI-TOF MS platform for clinical application is evidenced by the numerous tandem techniques that have been utilized with this form of mass spectrometry. These include MALDI combined with surface plasmon resonance and ELISA [39,40,41] as well as MALDI combined with traditional microscopy, imaging MALDI or MALDI mass spectrometry imaging (MSI). MALDI MSI has recently come to the forefront as a valuable tool for molecular pathology. In this technique a thin section of tissue can be mounted to a MALDI chip and molecular characterization of the tissue can be achieved through mass spectrometry. Coupled with traditional microscopy, MSI can identify protein, peptide and lipid content of the tissue without any *a priori* knowledge of its architecture [42,43,44].

In an Orbitrap detector, ions travel through a quadrupole and are passed to a central electrode where they orbit axially. Oscillations around this electrode are detected by Fourier Transform and produce very high resolution *m*/*z* assignments [45]. Hybrid quadrupole instruments combine the ion trapping capabilities of a triple quad with the high mass accuracy of the Orbitrap and are capable of accurately quantitating peptide mixtures after LC separation using parallel reaction monitoring (PRM). Unlike SRM, this method requires no a priori knowledge of the mass fragment transition, instead relying only upon the parent ion of interest and its elution time for post-acquisition data processing and quantification. This reduces the time required for method development and optimization, while providing higher specificity and selectivity than SRM analyses [46].

Traditionally, separation of analytes for ESI MS/MS relies upon reverse phase HPLC or UHPLC separation. Modern techniques allow for narrow elution peaks with reliable separation due to higher pressures and novel chromatographic substrates which provides increased selectivity and specificity for the mass analyzer. A recent approach for the characterization of amyloid fibrils utilized laser microdissection of formalin-fixed tissue sections and tandem mass spectrometry (ESI-MS/MS) to identify 94% of amyloid fibrils vs 76% identified by traditional methods [47]. This highlights the usefulness of combination methodologies for mass spectrometry in clinical settings.

### 2.3. Analyte Quantitation Methods

A major hurdle for the field of clinical proteomics is the development of unbiased, reproducible quantitation methodologies. In proteomics, there are two primary methods by which quantitative data is achieved: unlabeled and labeled [21,24,25]. Labeled methods to quantitate proteins include incorporation of a small mass tag, examples of which include isotope coded affinity tags (ICAT) and isobaric tags for relative and absolute quantitation (iTRAQ). Here, samples are treated with a low mass metabolic label unique for each sample analyzed and the samples are pooled prior to analysis. Using the distinct mass of each tag, the relative abundance of peptides can be compared between samples. Whereas the first commercially available system, ICAT, could be used to analyze differences between only two samples, the iTRAQ system is capable of multiplexing eight samples, greatly increasing the robustness of this technique. An alternative to this method is the addition of a stable isotope labeled internal standard for absolute quantitation (AQUA) [48]. Here a heavy peptide is digested which matches the protein digest of interest. This allows for the absolute quantitation of protein, with the drawback that it is cumbersome and requires a unique standard for each protein target. An AQUA alternative, Hi3, is based upon the observation that the concentration of protein in a complex mixture can be derived from the sum of the top three peptides ESI-MS signals. With the inclusion of a known standard, this method can be used to quantitate unfractionated lysates. Comparative studies between Hi3 and AQUA have shown that a single labeled internal standard is sufficient for global protein quantitation [34,49]. A recent advance in quantitative proteomics is the use of inductively coupled plasma mass spectrometry (ICP-MS) [50]. ICP-MS is an established method for metal detection used in quantitative proteomics either through the addition of metallic elemental tags which bind to specific functional groups within the protein, or through analyses of naturally occurring metals within proteins, the so called metallome. When coupled to LC-MS or MALDI-MS, ICP-MS provides a linear dynamic range of biomolecule detection that is orders of magnitude greater than those currently in use [49,51]. A thorough comparison of these methods is reviewed in [49].

Quantitation of targeted proteomics relies heavily upon SRM and MRM with an internal standard and requires complex statistical analyses. These methods have been slow to enter the clinical laboratory due to the time consuming validation for each mass transition. However, current work by a number of groups to build repositories for SRM mass transitions, e.g., Panorama [52], CPTAC [53], SRMatlas [54], and PASSEL [55], is likely to change that. In addition, a novel data independent approach, Sequential Windowed Acquisition of All Theoretical Fragment Ion Mass Spectra (SWATH-MS) [56], coupling a non-targeted acquisition with targeted analysis may provide a potential solution to the cumbersome nature of SRM and MRM. These developments bear close observation.

Because quantitation methods are only as good as the abundance of the analyte, enrichment is a critical step for all MS analyses. One method shown to increase detection sensitivity by several orders of magnitude is Stable Isotope Standards and Capture by Anti-Peptide Antibody (SISCAPA) [57]. SISCAPA combines inclusion of internal standards to account for variation in sample injection and digestion efficiency with enrichment of target protein by capture with anti-peptide antibodies. Nano-affinity columns are used to enrich specific peptides from digested protein samples spiked with labeled tryptic digests of the proteins of interest. This method highlights both the advantages and disadvantages of labeled quantitation. Low abundance peptides of interest can be enriched, resolved and accurately quantitated. The drawbacks are the need for the stable isotope labeled tryptic digests and the time required to develop methods specific to protein targets of interest. Affinity columns require the production of antibodies to all tryptic peptides used in quantification. Commercially available antibodies, although potentially suitable for this type of analysis, require extensive screening and have a low success rate [58]. The alternative to commercial antibodies are de novo generation which can be both time consuming and costly. An additional drawback is that only those peptides being sought at the beginning of the analysis can be quantified.

## 3. Personalized Proteomics

These advances in high throughput mass spectrometry of clinical samples allowed for the publication of a draft of the human proteome [59,60,61]. This collection of data from various tissues of healthy samples forms the basis for comparative clinical proteomics. By comparison of patient samples with this database, scientists associate changes in the proteomes with particular disease states. Translation of these findings into the clinic and hospital setting allows doctors the possibility of mass spectral analysis of patient proteomes. While initial comparison must be made between the patient sample and the database average, as bioinformatics and information processing grows, ultimately physicians will be able to compare an ill patient’s proteome with their own healthy archived records [62]. This Personalized Proteomic analysis has the potential to greatly increase the diagnostic accuracy while reducing time and costs.

Through rapid proteomic screening of easily accessible tissue samples, early determination of the presence and/or severity of disease allows for quick response from medical personnel and improved patient outcome. While this is certainly useful for biomarker monitoring, it places strong technological and monetary constraints on a burgeoning medical field. The power of proteomics is in discovering the meaningful unknown in a bouillabaisse of unrelated molecules with high mass accuracy and sensitivity. For this reason, clinical proteomics will be most useful for diagnosis of diseases with rare or unknown etiology, monitoring the therapeutic effect and activity of drug regimens, improving treatment options for individual patients, and, as always, discovering a brave new world of medical possibilities. Comparisons between proteomic analysis and immunoassays have been discussed elsewhere [24,32,63,64,65,66,67,68,69,70,71,72,73,74,75,76,77,78,79,80,81]; hence, this review will focus on the uses of proteomics in the clinical setting.

### 3.1. Proteomics for Biomarker Monitoring

Much of the current research directing the use of proteomics in the clinic or hospital is for the identification of biomarkers, though it largely remains in the discovery phase. The search for biomarkers can largely be broken down into two categories: those specific for the diagnosis of illness and those associated with disease severity. Ongoing research has already identified biomarkers of varying reliability for numerous cancers, intestinal bowel disease, amyotrophic lateral sclerosis, and other diseases [82,83,84,85].

Methodologies to quantitate biomarkers other than by mass spectrometry have demonstrated great success in the clinic. However, the limitations of these alternative strategies are namely two-fold: the number of analytes and its sensitivity. Current clinical assays to quantitate biomarkers generally analyze only a single molecule at a time. Complicated diagnoses may require analysis of multiple biomarkers to either rule in or rule out various diseases, taking precious time. In contrast, analysis of biomarkers using mass spectrometry can quantitate numerous biomarkers from a single sample and in a single test [86]. Multiplexing the analysis of a patient sample could significantly reduce the time required for a patient’s diagnosis. Several biomarkers detectable by mass spectrometry have already been associated with cardiac events (trimethylamine-N-oxide, MMP-2 MMP-9, MMP10, NGAL) [87,88,89,90,91,92], intestinal bowel disease [83], and numerous targets for various cancers [84,85,86,93,94,95,96,97].

Most known biomarkers come from proteins present at high levels in the serum or tissue, e.g., albumin, CRP, OVA1, etc. The challenge for precision medicine is to develop new biomarkers making use of the exquisite sensitivity of mass spectrometry for detection of biomarkers at lower abundance. These biomarkers may not necessarily be levels of protein expression but rather may include posttranslational modifications (PTMs), metabolites, or metabolic flux. In addition, identifying the appropriate molecular isoform from a complex mixture is necessary for several existing biomarkers. The sensitivity of mass spectrometry in the detection of addition of a phosphate, fucosyl group, ubiquitin, or glycosylation sumoylation, etc. makes mass spectrometry ideal for determining the relative abundance of these isoforms in disease association.

Many proteins exist in multiple states, either post-translationally modified or enzymatically cleaved. These PTMs and cleavages can be responsible for activation of the protein resulting in the disease state, or removal/inactivation of the protein causing the same. Therefore, the relative abundance of the precursor and processed forms of these proteins indicate disease or the predisposition for developing disease. For example, bioactive insulin is synthesized as preproinsulin containing a secretory signal peptide and an extraneous intramolecular fragment, C-peptide. Following removal of the signal peptides, proinsulin is packaged into secretory vesicles and the C-peptide is enzymatically excised from the interior of the proinsulin [98]. Thus, all three forms of the protein contain the sequence of bioactive insulin. Since the precursor and processed proteins share much sequence, and even structural, identity, determining the ratio of each can be difficult with immunoassays. However, proteomic technology is designed to determine the relative loss or gain of mass units [99,100]. In addition to insulin, growth hormone, parathyroid hormone, and albumin all exist as a heterogeneous mixture of various protein states with their relative abundances of potential clinical import [101,102,103,104]. Moreover, the aberrant production of a single clone of antibody is associated with several diseases, e.g., osteomyelitis, macroglobulinemia, and multiple myeloma. Tandem mass spectrometric sequencing of immunoglobulin chains can identify the overproduction of a given monoclonal antibody specificity amongst the numerous antibody specificities present at any given time in patient serum [105].

Furthermore, analysis of the proteomes of clinical specimens (tissues, bodily fluids, cultured primary cells) associated with a specific condition/illness can lead to the identification of novel biomarkers. Relative differences in the proteomes give an unbiased comparison between normal and affected samples. This untargeted approach to the discovery of novel biomarkers eliminates the need for *a priori* research or a systematic understanding of the condition before a biomarker can be identified. This untargeted, unbiased approach is particularly useful for new, rare or neglected diseases where little is understood about the physiology of the disease or the causative agent.

Another area where proteomics and mass spectrometry excel is the analysis of small molecule metabolites [106]. The presence and/or change of metabolite concentrations (metabolic flux) can signify an alteration in the normal functioning of a patient’s physiological state. Alterations in energy consumption and the ‘oncometabolite’ R-2-hydroxyglutarate have both been linked to cancer [107,108,109,110]. In theory, changes in a patient’s metabolites detected by mass spectrometry could indicate the presence of a tumor in a remote location not easily detectable by conventional means. Based on the specific metabolic flux, the treatment could therefore be adapted to the individual patient’s condition. However, this method of cancer diagnosis remains to be confirmed in a clinical setting.

### 3.2. Proteomics for Diagnosis

Rapid identification of an infectious disease remains challenging and time consuming often requiring multiple tests and even days of culture. Assemblage of databases containing the proteomes of humans and their pathogens for comparison to samples obtained in the clinic can result in immediate diagnosis of an infection [111]. Already, proteomic analysis can identify *E. coli*, *Salmonella*, *Campylobacter*, *Clostridium* (including *C. difficile*), *L. monocytogenes*, *Mycobacterium*, *Staphylococci*, *H. pylori*, and enterobacteriaceae from biological samples [112,113,114,115,116,117,118,119,120,121,122,123,124,125,126,127,128,129,130,131,132,133]. Many of these species contain multiple strains each encoding its individual combination of toxins and/or lethal factors. Isolate testing by proteomics has been reported to be faster and less costly for many of these infections [134,135,136]. Moreover, a single test can determine not only the bacterial strain but also antibiotic resistance mechanisms reliant upon alterations in protein expression [111,137,138].

Viral infections can be misdiagnosed as bacterial infections due to the use of clinical tests detecting levels of C-Reactive Protein (CRP) and IL-6 production, both of which are similarly upregulated in response to bacterial and viral infections, leading to prescription of ineffectual antibiotics for viral infection [139,140,141]. Rather, proteomic analysis of a patient sample can reveal both unique host proteins as well as specifically bacterial or viral proteins resulting in a more accurate diagnosis and treatment [142,143,144]. In addition, the success of a given therapeutic plan varies among patients being more or less effective for particular subpopulations of patients and for different stages of disease [145]. Therefore, monitoring the efficacy of antibiotics or antivirals after administration could quickly detect resistance or ineffectiveness of a particular treatment [143].

In addition, monitoring the processing, activation, and clearance of therapeutics could provide physicians with real time functional information about the success of the treatments administered (*in clinica* pharmacodynamics). Proteomic analysis of serial patient samples could potentially detect activation or breakdown of a therapeutic agent allowing physicians to monitor the therapeutic dose. Proteomic-based pharmacokinetic measurements of biotherapeutics have already been shown to be suitable alternatives to traditional assays [146,147,148,149]. Serial proteomic analysis could also monitor the progress, clearance, or lack thereof of a disease, e.g., infection or cancer, for each individual patient. As tumors are forced into remission, the altered metabolite profile discussed above should return to normal; conversely, proteomic analysis of tumors unresponsive to treatment would show no change. In addition, it was shown that the host peptidome, and likely the proteome from which these peptides were derived, changes in response to viral infection [18]. Therefore, even in autonomous transplant recipients, an infection in the host after transplant could alter the peptide repertoire presented by the transplant leading to rejection. Mass spectrometric monitoring for changes in the host peptidome following transplant could detect the potential for such viral-mediated peptidome shift and rejection, allowing for early intervention to tolerate the host to the new peptidome.

The detection of mass and mass losses by mass spectrometry also provides strong advantages for *in clinica* measurements of protein kinetics. This could be the activation of proteins through posttranslational modifications, as discussed above, but also rate of protein synthesis and clearance. Changes in these rates affect the overall concentration of target proteins, both host and/or biotherapeutic, which can inform dosing strategies. The high sensitivity and mass accuracy of the mass spectrometer makes it ideal for measurements of changes in these often low abundance proteins [150,151,152,153].

### 3.3. Proteomics to Improve Patient/Therapy Selection

The goal of precision medicine is to more specifically and reliably match the patient with the best course of therapy to ensure the optimal outcome for each individual patient. Just as blood group typing reduced complications with transfusions, genetic profiling of HLA loci has reduced transplant rejection rates by matching donor and recipient. Despite this level of scrutiny, as many as 10% of transplants result in rejection or graft versus host disease (GVHD) [154,155]. Differential alteration of the self and transplant peptidome after infection has been implicated as a potential cause for these complications [18]. Personalized proteomic analysis of the HLA loci, minor antigen loci, and peptide repertoire of potential donor/recipient pairs could reduce this rate even further [154,155,156]. Analysis at this level of specificity and detail requires the accuracy and sensitivity of mass spectrometry.

## 4. Conclusions

The use of proteomics in the clinic is the future of personalized medicine. However, the utility of personalized proteomics for diagnosis and treatment is dependent upon the meaningful translation of basic science research to medicine. The power of proteomics for use *in clinica* is also that which proves problematic for basic research. The translatability of basic research to the clinic has so far been limited due to the failure of biomarkers to apply to a large population. More stringency during the discovery phase with better verification of biomarkers and validation in preclinical studies are needed [21,25]. This is evident in the use of single cell proteomics in basic and applied research despite its limited utility *in clinica* as most conditions for which a patient might seek treatment involve the interaction of multiple cells and cell types and not the dysfunction of a single cell. Just because it is possible to generate a large amount of hypersensitive data mass accurate to four decimal places from a tiny amount of starting material does not necessarily mean this is useful to clinicians. Integration of basic and clinical research is needed to develop biomarkers and assays to definitively identify differential proteomics between healthy and diseased patient samples.

## Figures and Tables

**Table 1:** Comparison of diagnostic techniques.

Category	NGS/Genetics ^a^	ELISA ^b^	Flow Cytometry ^c^	Microarray ^d^	MS ^e^
Protein mutations	Only if encoded genetically	Sequence or structure recognized by antibody	Sequence or structure recognized by antibody & cellular	Only in sequence	Only in sequence
PTMs	Binding site may be identifiable	If differentially recognized by antibody	If differentially recognized by antibody & cellular	Reported for select PTMs	YES
Expression level	Inferred based on promoter/enhancers	YES	YES	YES	YES
Metabolites	Predicted	If unique antibody is available	If unique antibody is available & cellular	Selected	YES
Metabolic flux	Predicted	YES	Only intracellular	YES	YES
Enzymatic activity	Predicted	YES	No	Potential	YES
Number of analytes/test	Millions of bases	<100	15/40^f^	Thousands	Thousands
Targeted biomarkers	YES	YES	YES	YES	YES
Discovery analysis	YES	No	No	Limited	YES
Differentiate heterogeneous mixture	No	If differentially recognized by antibody	If differentially recognized by antibody & cellular	YES	YES
Serial analysis of patient samples	No new information is obtained	YES	YES	YES	YES
Pharmacokinetics	No	YES	No	Potential	YES
Therapeutic monitoring	No	YES	If cellular	Potential	YES

^a^ Next Generation Sequencing (NGS) and other genetic assays; ^b^ Enzyme-linked immunosorbent assay (ELISA) and other fluorescent/colorimetric antibody-based assays; ^c^ Flow cytometry, CyTOF, and imaging in flow assays; ^d^ DNA, RNA, protein, aptamer, antibody, small molecule, and peptide arrays; ^e^ Mass Spectrometry (MS) including proteomic, metabolomic and lipidomic assays and their derivatives; ^f^ 40 analytes is the current limit for CyTOF specifically.

## References

[1] Specific Genetic Disorders. https://www.genome.gov/10001204/specific-genetic-disorders/.

[2] Novelli G. (2010). Personalized genomic medicine. Intern. Emerg. Med..

[3] Jain K.K. (2002). Personalized medicine. Curr. Opin. Mol. Ther..

[4] Altsuler D., Donnelly P., Gibbs R.A., Yang H., Zeng C., Shen Y., Huang W., Wayne M.M.Y., Xue H., Chee M.S. (2005). The International HapMap Consortium. A haplotype map of the human genome. Nature.

[5] The Cost of Sequencing a Human Genome. https://www.genome.gov/27565109/the-cost-of-sequencing-a-human-genome/.

[6] Sudmant P.H., Rausch T., Gardner E.J., Handsaker R.E., Abyzov A., Huddleston J., Zhang Y., Ye K., Jun G., Hsi-Yang Fritz M. (2015). An integrated map of structural variation in 2504 human genomes. Nature.

[7] The 1000 Genomes Project Consortium (2015). A global reference for human genetic variation. Nature.

[8] The 1000 Genomes Project Consortium (2012). An integrated map of genetic variation from 1092 human genomes. Nature.

[9] The 1000 Genomes Project Consortium (2010). A map of human genome variation from population-scale sequencing. Nature.

[10] 1000 Genomes Project. http://www.1000genomes.org/data.

[11] Honey K. (2008). Gina: Making it safe to know what’s in your genes. J. Clin. Investig..

[12] ENCODE Project Consortium (2004). The ENCODE (ENCyclopedia of DNA Elements) Project. Science.

[13] Pennisi E. (2012). ENCODE project writes eulogy for junk DNA. Science.

[14] Roberts S., Julius M. (2016). Precision medicine: Now, not when. Healthc. Manag. Forum.

[15] Herberts C.A., van Gaans-van den Brink J., van der Heeft E., van Wijk M., Hoekman J., Jaye A., Poelen M.C.M., Boog C.J.P., Roholl P.J.M., Whittle H. (2003). Autoreactivity against induced or upregulated abundant self-peptides in HLA-A*0201 following measles virus infection. Hum. Immunol..

[16] Wahl A., Schafer F., Bardet W., Hildebrand W.H. (2010). HLA class I molecules reflect an altered host proteome after influenza virus infection. Hum. Immunol..

[17] Hickman H.D., Luis A.D., Bardet W., Buchli R., Battson C.L., Shearer M.H., Jackson K.W., Kennedy R.C., Hildebrand W.H. (2003). Cutting edge: Class I presentation of host peptides following HIV infection. J. Immunol..

[18] Spencer C.T., Bezbradica J.S., Ramos M.G., Arico C.D., Conant S.B., Gilchuk P., Gray J.J., Zheng M., Niu X., Hildebrand W. (2015). Viral infection causes a shift in the self peptide repertoire presented by human MHC class I molecules. Proteom. Clin. Appl..

[19] Gupta S., Manubhai K.P., Kulkarni V., Srivastava S. (2016). An overview of innovations and industrial solutions in protein microarray technology. Proteomics.

[20] Bandura D.R., Baranov V.I., Ornatsky O.I., Antonov A., Kinach R., Lou X., Pavlov S., Vorobiev S., Dick J.E., Tanner S.D. (2009). Mass cytometry: Technique for real time single cell multitarget immunoassay based on inductively coupled plasma time-of-flight mass spectrometry. Anal. Chem..

[21] Lehmann S., Brede C., Lescuyer P., Cocho J.A., Vialaret J., Bros P., Delatour V., Hirtz C. (2016). Clinical Mass Spectrometry Proteomics (CMSP) for medical laboratory: What does the future hold?. Chim. Acta Int. J. Clin. Chem..

[22] Lassman M.E., McAvoy T., Chappell D.L., Lee A.Y., Zhao X.X., Laterza O.F. (2016). The clinical utility of mass spectrometry based protein assays. Clin. Chim. Acta Int. J. Clin. Chem..

[23] Percy A.J., Byrns S., Pennington S.R., Holmes D.T., Anderson N.L., Agreste T.M., Duffy M.A. (2016). Clinical translation of MS-based, quantitative plasma proteomics: Status, challenges, requirements, and potential. Expert Rev. Proteom..

[24] Sabbagh B., Mindt S., Neumaier M., Findeisen P. (2016). Clinical applications of MS-based protein quantification. Proteom. Clin. Appl..

[25] Scherl A. (2015). Clinical protein mass spectrometry. Methods.

[26] Mischak H., Apweiler R., Banks R.E., Conaway M., Coon J., Dominiczak A., Ehrich J.H., Fliser D., Girolami M., Hermjakob H. (2007). Clinical proteomics: A need to define the field and to begin to set adequate standards. Proteom. Clin. Appl..

[27] Silva J.C., Gorenstein M.V., Li G.Z., Vissers J.P., Geromanos S.J. (2006). Absolute quantification of proteins by lcmse: A virtue of parallel MS acquisition. Mol. Cell. Proteom..

[28] Torsetnes S.B., Levernaes M.S., Broughton M.N., Paus E., Halvorsen T.G., Reubsaet L. (2014). Multiplexing determination of small cell lung cancer biomarkers and their isovariants in serum by immunocapture LC-MS/MS. Anal. Chem..

[29] Shi T., Fillmore T.L., Gao Y., Zhao R., He J., Schepmoes A.A., Nicora C.D., Wu C., Chambers J.L., Moore R.J. (2013). Long-gradient separations coupled with selected reaction monitoring for highly sensitive, large scale targeted protein quantification in a single analysis. Anal. Chem..

[30] Yamashita M., Fenn J.B. (1984). Electrospray ion source. Another variation on the free-jet theme. J. Phys. Chem..

[31] Tanaka K., Waki H., Ido Y., Akita S., Yoshida Y., Yoshida T., Matsuo T. (1988). Protein and polymer analyses up to *m*/*z* 100,000 by laser ionization time-of-flight mass spectrometry. Rapid Commun. Mass Spectrom..

[32] Addona T.A., Abbatiello S.E., Schilling B., Skates S.J., Mani D.R., Bunk D.M., Spiegelman C.H., Zimmerman L.J., Ham A.J., Keshishian H. (2009). Multi-site assessment of the precision and reproducibility of multiple reaction monitoring-based measurements of proteins in plasma. Nat. Biotechnol..

[33] Domanski D., Percy A.J., Yang J., Chambers A.G., Hill J.S., Freue G.V., Borchers C.H. (2012). MRM-based multiplexed quantitation of 67 putative cardiovascular disease biomarkers in human plasma. Proteomics.

[34] Kirkpatrick D.S., Gerber S.A., Gygi S.P. (2005). The absolute quantification strategy: A general procedure for the quantification of proteins and post-translational modifications. Methods.

[35] Jeudy J., Salvador A., Simon R., Jaffuel A., Fonbonne C., Leonard J.F., Gautier J.C., Pasquier O., Lemoine J. (2014). Overcoming biofluid protein complexity during targeted mass spectrometry detection and quantification of protein biomarkers by mrm cubed (MRM3). Anal. Bioanal. Chem..

[36] Geiger T., Cox J., Mann M. (2010). Proteomics on an orbitrap benchtop mass spectrometer using all-ion fragmentation. Mol. Cell. Proteom..

[37] Michalski A., Damoc E., Lange O., Denisov E., Nolting D., Muller M., Viner R., Schwartz J., Remes P., Belford M. (2012). Ultra high resolution linear ion trap orbitrap mass spectrometer (orbitrap elite) facilitates top down LC-MS/MS and versatile peptide fragmentation modes. Mol. Cell. Proteom..

[38] Dillen L., Cools W., Vereyken L., Lorreyne W., Huybrechts T., de Vries R., Ghobarah H., Cuyckens F. (2012). Comparison of triple quadrupole and high-resolution TOF-MS for quantification of peptides. Bioanalysis.

[39] Remy-Martin F., El Osta M., Lucchi G., Zeggari R., Leblois T., Bellon S., Ducoroy P., Boireau W. (2012). Surface plasmon resonance imaging in arrays coupled with mass spectrometry (Supra-MS): Proof of concept of on-chip characterization of a potential breast cancer marker in human plasma. Anal. Bioanal. Chem..

[40] Rouleau A., El Osta M., Lucchi G., Ducoroy P., Boireau W. (2012). Immuno-MALDI-MS in human plasma and on-chip biomarker characterizations at the femtomole level. Sensors.

[41] Trenchevska O., Kamcheva E., Nedelkov D. (2010). Mass spectrometric immunoassay for quantitative determination of protein biomarker isoforms. J. Proteome Res..

[42] Willems S.M., van Remoortere A., van Zeijl R., Deelder A.M., McDonnell L.A., Hogendoorn P.C. (2010). Imaging mass spectrometry of myxoid sarcomas identifies proteins and lipids specific to tumour type and grade, and reveals biochemical intratumour heterogeneity. J. Pathol..

[43] Meding S., Martin K., Gustafsson O.J., Eddes J.S., Hack S., Oehler M.K., Hoffmann P. (2013). Tryptic peptide reference data sets for MALDI imaging mass spectrometry on formalin-fixed ovarian cancer tissues. J. Proteome Res..

[44] Heijs B., Abdelmoula W.M., Lou S., Briaire-de Bruijn I.H., Dijkstra J., Bovee J.V., McDonnell L.A. (2015). Histology-guided high-resolution matrix-assisted laser desorption ionization mass spectrometry imaging. Anal. Chem..

[45] Makarov A. (2000). Electrostatic axially harmonic orbital trapping: A high-performance technique of mass analysis. Anal. Chem..

[46] Gallien S., Domon B. (2015). Advances in high-resolution quantitative proteomics: Implications for clinical applications. Expert Rev. Proteom..

[47] Gilbertson J.A., Theis J.D., Vrana J.A., Lachmann H., Wechalekar A., Whelan C., Hawkins P.N., Dogan A., Gillmore J.D. (2015). A comparison of immunohistochemistry and mass spectrometry for determining the amyloid fibril protein from formalin-fixed biopsy tissue. J. Clin. Pathol..

[48] Gerber S.A., Rush J., Stemman O., Kirschner M.W., Gygi S.P. (2003). Absolute quantification of proteins and phosphoproteins from cell lysates by tandem MS. Proc. Natl. Acad. Sci. USA.

[49] Muntel J., Fromion V., Goelzer A., Maabeta S., Mader U., Buttner K., Hecker M., Becher D. (2014). Comprehensive absolute quantification of the cytosolic proteome of bacillus subtilis by data independent, parallel fragmentation in liquid chromatography/mass spectrometry (LC/MS(e)). Mol. Cell. Proteom..

[50] Chahrour O., Cobice D., Malone J. (2015). Stable isotope labelling methods in mass spectrometry-based quantitative proteomics. J. Pharm. Biomed. Anal..

[51] Kretschy D., Koellensperger G., Hann S. (2012). Elemental labelling combined with liquid chromatography inductively coupled plasma mass spectrometry for quantification of biomolecules: A review. Anal. Chim. Acta.

[52] Sharma V., Eckels J., Taylor G.K., Shulman N.J., Stergachis A.B., Joyner S.A., Yan P., Whiteaker J.R., Halusa G.N., Schilling B. (2014). Panorama: A targeted proteomics knowledge base. J. Proteome Res..

[53] Whiteaker J.R., Halusa G.N., Hoofnagle A.N., Sharma V., MacLean B., Yan P., Wrobel J.A., Kennedy J., Mani D.R., Zimmerman L.J. (2014). CPTAC assay portal: A repository of targeted proteomic assays. Nat. Methods.

[54] Kusebauch U., Campbell D.S., Deutsch E.W., Chu C.S., Spicer D.A., Brusniak M.Y., Slagel J., Sun Z., Stevens J., Grimes B. (2016). Human SRMAtlas: A resource of targeted assays to quantify the complete human proteome. Cell.

[55] Farrah T., Deutsch E.W., Kreisberg R., Sun Z., Campbell D.S., Mendoza L., Kusebauch U., Brusniak M.-Y., Hüttenhain R., Schiess R. (2012). Passel: The peptideatlas SRMExperiment library. Proteomics.

[56] Gillet L.C., Navarro P., Tate S., Röst H., Selevsek N., Reiter L., Bonner R., Aebersold R. (2012). Targeted data extraction of the MS/MS spectra generated by data-independent acquisition: A new concept for consistent and accurate proteome analysis. Mol. Cell. Proteom..

[57] Anderson N.L., Anderson N.G., Haines L.R., Hardie D.B., Olafson R.W., Pearson T.W. (2004). Mass spectrometric quantitation of peptides and proteins using stable isotope standards and capture by anti-peptide antibodies (SISCAPA). J. Proteome Res..

[58] Schoenherr R.M., Zhao L., Ivey R.G., Voytovich U.J., Kennedy J., Yan P., Lin C., Whiteaker J.R., Paulovich A.G. (2016). Commercially available antibodies can be applied in quantitative multiplexed peptide immunoaffinity enrichment targeted mass spectrometry assays. Proteomics.

[59] Uhlen M., Fagerberg L., Hallstrom B.M., Lindskog C., Oksvold P., Mardinoglu A., Sivertsson A., Kampf C., Sjostedt E., Asplund A. (2015). Proteomics. Tissue-based map of the human proteome. Science.

[60] Wilhelm M., Schlegl J., Hahne H., Moghaddas Gholami A., Lieberenz M., Savitski M.M., Ziegler E., Butzmann L., Gessulat S., Marx H. (2014). Mass-spectrometry-based draft of the human proteome. Nature.

[61] Kim M.S., Pinto S.M., Getnet D., Nirujogi R.S., Manda S.S., Chaerkady R., Madugundu A.K., Kelkar D.S., Isserlin R., Jain S. (2014). A draft map of the human proteome. Nature.

[62] Lindskog C. (2015). The potential clinical impact of the tissue-based map of the human proteome. Expert Rev. Proteom..

[63] Prakash A., Rezai T., Krastins B., Sarracino D., Athanas M., Russo P., Zhang H., Tian Y., Li Y., Kulasingam V. (2012). Interlaboratory reproducibility of selective reaction monitoring assays using multiple upfront analyte enrichment strategies. J. Proteome Res..

[64] Prakash A., Rezai T., Krastins B., Sarracino D., Athanas M., Russo P., Ross M.M., Zhang H., Tian Y., Kulasingam V. (2010). Platform for establishing interlaboratory reproducibility of selected reaction monitoring-based mass spectrometry peptide assays. J. Proteome Res..

[65] Abbatiello S.E., Schilling B., Mani D.R., Zimmerman L.J., Hall S.C., MacLean B., Albertolle M., Allen S., Burgess M., Cusack M.P. (2015). Large-scale interlaboratory study to develop, analytically validate and apply highly multiplexed, quantitative peptide assays to measure cancer-relevant proteins in plasma. Mol. Cell. Proteom..

[66] Hoofnagle A.N., Becker J.O., Oda M.N., Cavigiolio G., Mayer P., Vaisar T. (2012). Multiple-reaction monitoring–mass spectrometric assays can accurately measure the relative protein abundance in complex mixtures. Clin. Chem..

[67] Shi T., Sun X., Gao Y., Fillmore T.L., Schepmoes A.A., Zhao R., He J., Moore R.J., Kagan J., Rodland K.D. (2013). Targeted quantification of low ng/mL level proteins in human serum without immunoaffinity depletion. J. Proteome Res..

[68] Shi T., Su D., Liu T., Tang K., Camp D.G., Qian W.-J., Smith R.D. (2012). Advancing the sensitivity of selected reaction monitoring-based targeted quantitative proteomics. Proteomics.

[69] Rauh M. (2012). LC-MS/MS for protein and peptide quantification in clinical chemistry. J. Chromatogr. B.

[70] Nedelkov D. (2012). Mass spectrometry-based protein assays for in vitro diagnostic testing. Expert Rev. Mol. Diagn..

[71] Boja E.S., Rodriguez H. (2012). Mass spectrometry-based targeted quantitative proteomics: Achieving sensitive and reproducible detection of proteins. Proteomics.

[72] Fu Q., Schoenhoff F.S., Savage W.J., Zhang P., Van Eyk J.E. (2010). Multiplex assays for biomarker research and clinical application: Translational science coming of age. Proteom. Clin. Appl..

[73] Krastins B., Prakash A., Sarracino D.A., Nedelkov D., Niederkofler E.E., Kiernan U.A., Nelson R., Vogelsang M.S., Vadali G., Garces A. (2013). Rapid development of sensitive, high-throughput, quantitative and highly selective mass spectrometric targeted immunoassays for clinically important proteins in human plasma and serum. Clin. Biochem..

[74] Wu A.H.B., French D. (2013). Implementation of liquid chromatography/mass spectrometry into the clinical laboratory. Clin. Chim. Acta.

[75] Van Den Ouweland J.M.W., Kema I.P. (2012). The role of liquid chromatography–tandem mass spectrometry in the clinical laboratory. J. Chromatogr. B.

[76] Strathmann F.G., Hoofnagle A.N. (2011). Current and future applications of mass spectrometry to the clinical laboratory. Am. J. Clin. Pathol..

[77] Seger C. (2012). Usage and limitations of liquid chromatography-tandem mass spectrometry (LC-MS/MS) in clinical routine laboratories. Wien. Med. Wochenschr..

[78] Becker J.O., Hoofnagle A.N. (2012). Replacing immunoassays with tryptic digestion-peptide immunoaffinity enrichment and LC-MS/MS. Bioanalysis.

[79] Hoofnagle A.N., Wener M.H. (2009). The fundamental flaws of immunoassays and potential solutions using tandem mass spectrometry. J. Immunol. Methods.

[80] Trenchevska O., Schaab M.R., Nelson R.W., Nedelkov D. (2015). Development of multiplex mass spectrometric immunoassay for detection and quantification of apolipoproteins C-I, C-II, C-III and their proteoforms. Methods.

[81] Vilà-Rico M., Colomé-Calls N., Martín-Castel L., Gay M., Azorín S., Vilaseca M., Planas A., Canals F. (2015). Quantitative analysis of post-translational modifications in human serum transthyretin associated with familial amyloidotic polyneuropathy by targeted LC-MS and intact protein MS. J. Proteom..

[82] Chen Y., Liu X.H., Wu J.J., Ren H.M., Wang J., Ding Z.T., Jiang Y.P. (2016). Proteomic analysis of cerebrospinal fluid in amyotrophic lateral sclerosis. Exp. Ther. Med..

[83] Chan P.P., Wasinger V.C., Leong R.W. (2016). Current application of proteomics in biomarker discovery for inflammatory bowel disease. World J. Gastrointest. Pathophysiol..

[84] Bandu R., Mok H.J., Kim K.P. (2016). Phospholipids as cancer biomarkers: Mass spectrometry-based analysis. Mass Spectrom. Rev..

[85] Di Meo A., Pasic M.D., Yousef G.M. (2016). Proteomics and peptidomics: Moving toward precision medicine in urological malignancies. Oncotarget.

[86] Wang H., Hanash S. (2015). Mass spectrometry based proteomics for absolute quantification of proteins from tumor cells. Methods.

[87] Tang W.H., Wang Z., Levison B.S., Koeth R.A., Britt E.B., Fu X., Wu Y., Hazen S.L. (2013). Intestinal microbial metabolism of phosphatidylcholine and cardiovascular risk. N. Engl. J. Med..

[88] Tang W.H., Wang Z., Kennedy D.J., Wu Y., Buffa J.A., Agatisa-Boyle B., Li X.S., Levison B.S., Hazen S.L. (2015). Gut microbiota-dependent trimethylamine N-oxide (TMAO) pathway contributes to both development of renal insufficiency and mortality risk in chronic kidney disease. Circ. Res..

[89] Friese R.S., Rao F., Khandrika S., Thomas B., Ziegler M.G., Schmid-Schonbein G.W., O’Connor D.T. (2009). Matrix metalloproteinases: Discrete elevations in essential hypertension and hypertensive end-stage renal disease. Clin. Exp. Hypertens..

[90] Hobeika M.J., Thompson R.W., Muhs B.E., Brooks P.C., Gagne P.J. (2007). Matrix metalloproteinases in peripheral vascular disease. J. Vasc. Surg..

[91] Daniels L.B., Barrett-Connor E., Clopton P., Laughlin G.A., Ix J.H., Maisel A.S. (2012). Plasma neutrophil gelatinase-associated lipocalin is independently associated with cardiovascular disease and mortality in community-dwelling older adults: The rancho bernardo study. J. Am. Coll. Cardiol..

[92] De Franciscis S., Serra R. (2015). Matrix metalloproteinases and endothelial dysfunction: The search for new prognostic markers and for new therapeutic targets for vascular wall imbalance. Thromb. Res..

[93] Srinivas P.R., Verma M., Zhao Y., Srivastava S. (2002). Proteomics for cancer biomarker discovery. Clin. Chem..

[94] Longoria T.C., Ueland F.R., Zhang Z., Chan D.W., Smith A., Fung E.T., Munroe D.G., Bristow R.E. (2014). Clinical performance of a multivariate index assay for detecting early-stage ovarian cancer. Am. J. Obstet. Gynecol..

[95] Sreekumar A., Poisson L.M., Rajendiran T.M., Khan A.P., Cao Q., Yu J., Laxman B., Mehra R., Lonigro R.J., Li Y. (2009). Metabolomic profiles delineate potential role for sarcosine in prostate cancer progression. Nature.

[96] Cernei N., Heger Z., Gumulec J., Zitka O., Masarik M., Babula P., Eckschlager T., Stiborova M., Kizek R., Adam V. (2013). Sarcosine as a potential prostate cancer biomarker—A review. Int. J. Mol. Sci..

[97] Xu W., Yang H., Liu Y., Yang Y., Wang P., Kim S.H., Ito S., Yang C., Wang P., Xiao M.T. (2011). Oncometabolite 2-hydroxyglutarate is a competitive inhibitor of alpha-ketoglutarate-dependent dioxygenases. Cancer Cell.

[98] Fu Z., Gilbert E.R., Liu D. (2013). Regulation of insulin synthesis and secretion and pancreatic beta-cell dysfunction in diabetes. Curr. Diabetes Rev..

[99] Kippen A.D., Cerini F., Vadas L., Stöcklin R., Vu L., Offord R.E., Rose K. (1997). Development of an isotope dilution assay for precise determination of insulin, c-peptide, and proinsulin levels in non-diabetic and type ii diabetic individuals with comparison to immunoassay. J. Biol. Chem..

[100] Zhang Y., Fonslow B.R., Shan B., Baek M.C., Yates J.R. (2013). Protein analysis by shotgun/bottom-up proteomics. Chem. Rev..

[101] Kushnir M.M., Rockwood A.L., Strathmann F.G., Frank E.L., Straseski J.A., Meikle A.W. (2016). LC-MS/MS measurement of parathyroid hormone–related peptide. Clin. Chem..

[102] Wieringa G.E., Sturgeon C.M., Trainer P.J. (2014). The harmonisation of growth hormone measurements: Taking the next steps. Clin. Chim. Acta.

[103] Arsene C.G., Kratzsch J., Henrion A. (2014). Mass spectrometry—An alternative in growth hormone measurement. Bioanalysis.

[104] Miller W.G., Bruns D.E., Hortin G.L., Sandberg S., Aakre K.M., McQueen M.J., Itoh Y., Lieske J.C., Seccombe D.W., Jones G. (2009). Current issues in measurement and reporting of urinary albumin excretion. Clin. Chem..

[105] Mills J.R., Barnidge D.R., Murray D.L. (2015). Detecting monoclonal immunoglobulins in human serum using mass spectrometry. Methods.

[106] Stringer K.A., McKay R.T., Karnovsky A., Quemerais B., Lacy P. (2016). Metabolomics and its application to acute lung diseases. Front. Immunol..

[107] Vander Heiden M.G., Cantley L.C., Thompson C.B. (2009). Understanding the warburg effect: The metabolic requirements of cell proliferation. Science.

[108] Dang L., White D.W., Gross S., Bennett B.D., Bittinger M.A., Driggers E.M., Fantin V.R., Jang H.G., Jin S., Keenan M.C. (2009). Cancer-associated IDH1 mutations produce 2-hydroxyglutarate. Nature.

[109] Ward P.S., Patel J., Wise D.R., Abdel-Wahab O., Bennett B.D., Coller H.A., Cross J.R., Fantin V.R., Hedvat C.V., Perl A.E. (2010). The common feature of leukemia-associated IDH1 and IDH2 mutations is a neomorphic enzyme activity converting alpha-ketoglutarate to 2-hydroxyglutarate. Cancer Cell.

[110] Losman J.A., Kaelin W.G. (2013). What a difference a hydroxyl makes: Mutant IDH, (*R*)-2-hydroxyglutarate, and cancer. Genes Dev..

[111] Cheng K., Chui H., Domish L., Hernandez D., Wang G. (2016). Recent development of mass spectrometry and proteomics applications in identification and typing of bacteria. Proteom. Clin. Appl..

[112] Alispahic M., Christensen H., Bisgaard M., Hess M., Hess C. (2014). MALDI-TOF mass spectrometry confirms difficulties in separating species of the avibacterium genus. Avian Pathol..

[113] Samb-Ba B., Mazenot C., Gassama-Sow A., Dubourg G., Richet H., Hugon P., Lagier J.C., Raoult D., Fenollar F. (2014). MALDI-TOF identification of the human gut microbiome in people with and without diarrhea in senegal. PLoS ONE.

[114] Xiao D., Zhang H., He L., Peng X., Wang Y., Xue G., Su P., Zhang J. (2014). High natural variability bacteria identification and typing: Helicobacter pylori analysis based on peptide mass fingerprinting. J. Proteom..

[115] Kooken J., Fox K., Fox A., Altomare D., Creek K., Wunschel D., Pajares-Merino S., Martinez-Ballesteros I., Garaizar J., Oyarzabal O. (2014). Identification of staphylococcal species based on variations in protein sequences (mass spectrometry) and DNA sequence (soda microarray). Mol. Cell. Probes.

[116] Lasch P., Fleige C., Stammler M., Layer F., Nubel U., Witte W., Werner G. (2014). Insufficient discriminatory power of MALDI-TOF mass spectrometry for typing of enterococcus faecium and staphylococcus aureus isolates. J. Microbiol. Methods.

[117] Lima Ede O., de Macedo C.S., Esteves C.Z., de Oliveira D.N., Pessolani M.C., Nery J.A., Sarno E.N., Catharino R.R. (2015). Skin imprinting in silica plates: A potential diagnostic methodology for leprosy using high-resolution mass spectrometry. Anal. Chem..

[118] Barbuddhe S.B., Maier T., Schwarz G., Kostrzewa M., Hof H., Domann E., Chakraborty T., Hain T. (2008). Rapid identification and typing of listeria species by matrix-assisted laser desorption ionization-time of flight mass spectrometry. Appl. Environ. Microbiol..

[119] Moura H., Terilli R.R., Woolfitt A.R., Williamson Y.M., Wagner G., Blake T.A., Solano M.I., Barr J.R. (2013). Proteomic analysis and label-free quantification of the large clostridium difficile toxins. Int. J. Proteom..

[120] Wang D., Krilich J., Baudys J., Barr J.R., Kalb S.R. (2015). Enhanced detection of type C botulinum neurotoxin by the Endopep-MS assay through optimization of peptide substrates. Bioorg. Med. Chem..

[121] Kalb S.R., Baudys J., Wang D., Barr J.R. (2015). Recommended mass spectrometry-based strategies to identify botulinum neurotoxin-containing samples. Toxins.

[122] Grosse-Herrenthey A., Maier T., Gessler F., Schaumann R., Bohnel H., Kostrzewa M., Kruger M. (2008). Challenging the problem of clostridial identification with matrix-assisted laser desorption and ionization-time-of-flight mass spectrometry (MALDI-TOF MS). Anaerobe.

[123] McFarland M.A., Andrzejewski D., Musser S.M., Callahan J.H. (2014). Platform for identification of salmonella serovar differentiating bacterial proteins by top-down mass spectrometry: S. Typhimurium vs S. Heidelberg. Anal. Chem..

[124] Fagerquist C.K., Zaragoza W.J., Sultan O., Woo N., Quinones B., Cooley M.B., Mandrell R.E. (2014). Top-down proteomic identification of Shiga toxin 2 subtypes from Shiga toxin-producing *Escherichia coli* by matrix-assisted laser desorption ionization-tandem time of flight mass spectrometry. Appl. Environ. Microbiol..

[125] Cheng K., Sloan A., Peterson L., McCorrister S., Robinson A., Walker M., Drew T., McCrea J., Chui L., Wylie J. (2014). Comparative study of traditional flagellum serotyping and liquid chromatography-tandem mass spectrometry-based flagellum typing with clinical Escherichia coli isolates. J. Clin. Microbiol..

[126] Clark C.G., Kruczkiewicz P., Guan C., McCorrister S.J., Chong P., Wylie J., van Caeseele P., Tabor H.A., Snarr P., Gilmour M.W. (2013). Evaluation of MALDI-TOF mass spectroscopy methods for determination of Escherichia coli pathotypes. J. Microbiol. Methods.

[127] Kooken J., Fox K., Fox A., Wunschel D. (2014). Assessment of marker proteins identified in whole cell extracts for bacterial speciation using liquid chromatography electrospray ionization tandem mass spectrometry. Mol. Cell. Probes.

[128] Jung J.S., Popp C., Sparbier K., Lange C., Kostrzewa M., Schubert S. (2014). Evaluation of matrix-assisted laser desorption ionization-time of flight mass spectrometry for rapid detection of beta-lactam resistance in Enterobacteriaceae derived from blood cultures. J. Clin. Microbiol..

[129] Gekenidis M.T., Studer P., Wuthrich S., Brunisholz R., Drissner D. (2014). Beyond the matrix-assisted laser desorption ionization (MALDI) biotyping workflow: In search of microorganism-specific tryptic peptides enabling discrimination of subspecies. Appl. Environ. Microbiol..

[130] Chui H., Chan M., Hernandez D., Chong P., McCorrister S., Robinson A., Walker M., Peterson L.A., Ratnam S., Haldane D.J. (2015). Rapid, Sensitive, and Specific Escherichia coli H Antigen Typing by Matrix-Assisted Laser Desorption Ionization-Time of Flight-Based Peptide Mass Fingerprinting. J. Clin. Microbiol..

[131] Conway G.C., Smole S.C., Sarracino D.A., Arbeit R.D., Leopold P.E. (2001). Phyloproteomics: Species identification of Enterobacteriaceae using matrix-assisted laser desorption/ionization time-of-flight mass spectrometry. J. Mol. Microbiol. Biotechnol..

[132] Richter S.S., Sercia L., Branda J.A., Burnham C.A., Bythrow M., Ferraro M.J., Garner O.B., Ginocchio C.C., Jennemann R., Lewinski M.A. (2013). Identification of Enterobacteriaceae by matrix-assisted laser desorption/ionization time-of-flight mass spectrometry using the VITEK MS system. Eur. J. Clin. Microbiol. Infect. Dis..

[133] Zautner A.E., Masanta W.O., Tareen A.M., Weig M., Lugert R., Gross U., Bader O. (2013). Discrimination of multilocus sequence typing-based Campylobacter jejuni subgroups by MALDI-TOF mass spectrometry. BMC Microbiol..

[134] Segawa S., Sawai S., Murata S., Nishimura M., Beppu M., Sogawa K., Watanabe M., Satoh M., Matsutani T., Kobayashi M. (2014). Direct application of MALDI-TOF mass spectrometry to cerebrospinal fluid for rapid pathogen identification in a patient with bacterial meningitis. Clin. Chim. Acta Int. J. Clin. Chem..

[135] Nyvang Hartmeyer G., Kvistholm Jensen A., Bocher S., Damkjaer Bartels M., Pedersen M., Engell Clausen M., Abdul-Redha R., Dargis R., Schouenborg P., Hojlyng N. (2010). Mass spectrometry: Pneumococcal meningitis verified and Brucella species identified in less than half an hour. Scand. J. Infect. Dis..

[136] Angeletti S., Dicuonzo G., D’Agostino A., Avola A., Crea F., Palazzo C., Dedej E., De Florio L. (2015). Turnaround time of positive blood cultures after the introduction of matrix-assisted laser desorption-ionization time-of-flight mass spectrometry. New Microbiol..

[137] DeMarco M.L., Ford B.A. (2013). Beyond identification: Emerging and future uses for MALDI-TOF mass spectrometry in the clinical microbiology laboratory. Clin. Lab. Med..

[138] March G.A., Garcia-Loygorri M.C., Simarro M., Gutierrez M.P., Orduna A., Bratos M.A. (2015). A new approach to determine the susceptibility of bacteria to antibiotics directly from positive blood culture bottles in two hours. J. Microbiol. Methods.

[139] Craig J.C., Williams G.J., Jones M., Codarini M., Macaskill P., Hayen A., Irwig L., Fitzgerald D.A., Isaacs D., McCaskill M. (2010). The accuracy of clinical symptoms and signs for the diagnosis of serious bacterial infection in young febrile children: Prospective cohort study of 15,781 febrile illnesses. BMJ.

[140] Van den Bruel A., Thompson M.J., Haj-Hassan T., Stevens R., Moll H., Lakhanpaul M., Mant D. (2011). Diagnostic value of laboratory tests in identifying serious infections in febrile children: Systematic review. BMJ.

[141] Laxminarayan R., Duse A., Wattal C., Zaidi A.K., Wertheim H.F., Sumpradit N., Vlieghe E., Hara G.L., Gould I.M., Goossens H. (2013). Antibiotic resistance-the need for global solutions. Lancet Infect. Dis..

[142] Oved K., Cohen A., Boico O., Navon R., Friedman T., Etshtein L., Kriger O., Bamberger E., Fonar Y., Yacobov R. (2015). A novel host-proteome signature for distinguishing between acute bacterial and viral infections. PLoS ONE.

[143] Valenzuela-Sanchez F., Valenzuela-Mendez B., Rodriguez-Gutierrez J.F., Rello J. (2016). Personalized medicine in severe influenza. Eur. J. Clin. Microbiol. Infect. Dis..

[144] Schuetz P., Hausfater P., Amin D., Amin A., Haubitz S., Faessler L., Kutz A., Conca A., Reutlinger B., Canavaggio P. (2015). Biomarkers from distinct biological pathways improve early risk stratification in medical emergency patients: The multinational, prospective, observational TRIAGE study. Crit. Care.

[145] Collins F.S., Varmus H. (2015). A new initiative on precision medicine. New Engl. J. Med..

[146] Ezan E., Dubois M., Becher F. (2009). Bioanalysis of recombinant proteins and antibodies by mass spectrometry. Analyst.

[147] An B., Zhang M., Qu J. (2014). Toward Sensitive and Accurate Analysis of Antibody Biotherapeutics by Liquid Chromatography Coupled with Mass Spectrometry. Drug Metabol. Dispos..

[148] Zheng J., Mehl J., Zhu Y., Xin B., Olah T. (2014). Application and challenges in using LC-MS assays for absolute quantitative analysis of therapeutic proteins in drug discovery. Bioanalysis.

[149] Van Den Broek I., Niessen W.M.A., van Dongen W.D. (2013). Bioanalytical LC-MS/MS of protein-based biopharmaceuticals. J. Chromatogr. B.

[150] Lassman M.E., McAvoy T., Lee A.Y., Chappell D., Wong O., Zhou H., Reyes-Soffer G., Ginsberg H.N., Millar J.S., Rader D.J. (2014). Practical immunoaffinity-enrichment LC-MS for measuring protein kinetics of low-abundance proteins. Clin. Chem..

[151] Millar J.S., Reyes-Soffer G., Jumes P., Dunbar R.L., deGoma E.M., Baer A.L., Karmally W., Donovan D.S., Rafeek H., Pollan L. (2015). Anacetrapib lowers LDL by increasing ApoB clearance in mildly hypercholesterolemic subjects. J. Clin. Investig..

[152] Bateman R.J., Munsell L.Y., Morris J.C., Swarm R., Yarasheski K.E., Holtzman D.M. (2006). Human amyloid-beta synthesis and clearance rates as measured in cerebrospinal fluid in vivo. Nat. Med..

[153] Zhou H., Castro-Perez J., Lassman M.E., Thomas T., Li W., McLaughlin T., Dan X., Jumes P., Wagner J.A., Gutstein D.E. (2013). Measurement of apo(a) kinetics in human subjects using a microfluidic device with tandem mass spectrometry. Rapid Commun. Mass Spectrom..

[154] Gratwohl A., Dohler B., Stern M., Opelz G. (2008). H-Y as a minor histocompatibility antigen in kidney transplantation: A retrospective cohort study. Lancet.

[155] Dierselhuis M., Goulmy E. (2009). The relevance of minor histocompatibility antigens in solid organ transplantation. Curr. Opin. Organ Transplant..

[156] Spencer C.T., Gilchuk P., Dragovic S.M., Joyce S. (2010). Minor histocompatibility antigens: Presentation principles, recognition logic and the potential for a healing hand. Curr. Opin. Organ Transplant..

